# ABCA1: A Therapeutic Target for Improving Cholesterol Homeostasis in Peripheral Neuropathies

**DOI:** 10.3390/biom16020332

**Published:** 2026-02-22

**Authors:** Yeon Hwa Woo, Natalie E. Schmidt, Jan O. Johansson, Lucia Notterpek

**Affiliations:** 1 Department of Physiology and Cell Biology, School of Medicine, University of Nevada, Reno, NV 89557, USA; yeonw88@gmail.com (Y.H.W.); natalieschmidt@med.unr.edu (N.E.S.); 2 Artery Therapeutics, Inc., 10 Duberstein Dr, Suite A, San Ramon, CA 94583, USA; jjohansson@arterytx.com

**Keywords:** ATP-binding cassette A1 (ABCA1), cholesterol transport, Charcot-Marie-Tooth (CMT) disease, peripheral myelin protein 22 (PMP22), Tangier disease, peripheral neuropathy, myelination, myelinating glia, Schwann

## Abstract

ATP-binding cassette A1 (ABCA1) is a critical molecule in facilitating cholesterol transport in a variety of organs. In the nervous system, cholesterol supply is essential and rate-limiting for myelin biogenesis, which underlies efficient conduction of nerve impulses. When myelin is damaged or improperly formed due to genetic defects, a host of neurological symptoms may arise. A rare form of peripheral neuropathy in Tangier disease (TD) patients is associated with autosomal recessive mutations in ABCA1. Accordingly, when ABCA1 loses its function due to misexpression, the neuropathic phenotype is over-represented. Independently, studies have revealed the altered expression of ABCA1 and dysregulation of cholesterol metabolism in a host of inherited peripheral neuropathies engaging the Peripheral Myelin Protein 22 (PMP22), suggesting shared pathophysiology. While the role of ABCA1 has not been investigated broadly in peripheral nerves, the transporter molecule is a therapeutic target for human disorders, including multiple sclerosis and Alzheimer’s disease. Investigations in rodent models of type 1 Charcot–Marie–Tooth (CMT) neuropathies support the candidacy of this cholesterol transporter as a therapeutic target in efforts of peripheral myelin repair. Ongoing preclinical studies in central and peripheral nervous system disease models will provide critical information on the importance of ABCA1 as a target for disease modifying intervention.

## 1. Introduction

Peripheral neuropathies comprise a heterogeneous group of disorders that can result from genetic mutations or be associated with comorbid conditions. Inherited forms are progressive and have been linked to mutations in over 100 genes expressed in axons, myelinating glia, or both [1]. The most common type of hereditary motor and sensory neuropathies are Charcot–Marie–Tooth (CMT) diseases, which are further classified as type 1 and type 2 [2]. In type 2 CMTs, the primary pathology initiates in the nerve axons, while in type 1 CMTs the myelin producing glial cells, termed Schwann cells, are affected [3]. Patients suffering from neuropathies may have muscle weakness, sensory loss and foot deformities, and variable abnormalities in the speed of nerve signal propagation. Myelin, the primary site of pathology in type 1 CMTs, is a lipid-rich sheath, synthesized by Schwann cells, that serves to insulate neuronal axons, thereby increasing the speed and accuracy of signal transduction. In early-onset type 1 CMTs, myelin synthesis is disrupted during development (dys-myelination), while in other neuropathies myelin degradation may occur later in life (demyelination) [4]. Even though the genetic linkages for many neuropathies are now recognized, the mechanisms by which these defects cause disease are not fully understood. One common disturbed pathway in a variety of neuropathies is variations in lipid metabolism [5]. Given that lipids comprise about half of the human brain’s weight and 80% of myelin’s dry weight, this is not surprising. Among the various organelles and membranes found in neurons and glia, myelin is particularly enriched in neutral lipids, ensuring high packing density, membrane stability, and reduced permeability to facilitate nerve impulse conduction [6]. Cholesterol is the predominant neutral lipid of myelin, and its availability is rate-limiting for myelin synthesis in the CNS and PNS [7]. Regulation of cholesterol transport in myelin-forming oligodendrocytes and Schwann cells is therefore of fundamental importance.

In the CNS, cholesterol is primarily synthesized by astrocytes and delivered to neurons and oligodendrocytes through the Apolipoprotein E (ApoE)-mediated pathway whereas in the PNS, myelinating Schwann cells are the primary source of cholesterol for myelin production [8,9,10]. During peripheral nerve injury and subsequent regeneration, Schwann cells respond by recycling cholesterol and lipids through low-density lipoprotein receptor (LDLR)-mediated uptake [11], or by increased cholesterol synthesis. Critical mediators of peripheral nerve and myelin regeneration include several lipid metabolism-related molecules, including LDLR, ApoE, Apolipoprotein D and cholesterol [12,13,14]. ATP-binding cassette A1 (ABCA1) is the major cholesterol carrier in the CNS that facilitates cholesterol transport to ApoE [15]; however, its role in the PNS has not been studied in detail. Yet, altered expression of ApoE, particularly the E4 allele, and of ABCA1, independently, have been associated with peripheral nerve dysfunction [16,17].

Given the well-established regenerative capacity of the PNS in response to acute injury, and the role of lipid metabolism in this process, it is plausible that nerve damage caused by inherited gene defects, such as those linked with CMT diseases, may also be amenable to repair. The expression of ABCA1 is increased 4-fold after nerve injury, and cells deficient in ABCA1 are compromised in their ability to extend neurites [18]. Notably, nerve injury did not increase de novo cholesterol synthesis, suggesting that the cells recycled lipids during the repair process. Yet little is known about the role of cholesterol transport proteins such as ABCA1 in the pathophysiology of chronic, life-long inherited peripheral neuropathies, a topic that is the focus of this review article. Understanding how cholesterol is transported within Schwann cells during myelin formation and repair will provide critical knowledge for the identification and validation of new therapeutic targets in inherited demyelinating neuropathies. For a comprehensive review of the alterations in lipid metabolism in peripheral neuropathies please see a recent review by Silva and colleagues [5]. Further, the translational significance of cholesterol-based therapies in neurological disorders of the CNS was also recently reviewed and will not be extensively covered here [19].

## 2. ABCA1 and ABCA2 in the Nervous System

ABCA1 is a member of the ATP-binding cassette (ABC) family of membrane transporters, which constitute one of the largest groups of cholesterol and phospholipid transporters across the plasma membrane [20]. ABCA1 has six transmembrane, two extracellular binding domains, and two intracellular nucleotide-binding domains with an intervening hydrophobic loop [21]. In the CNS, ABCA1 is expressed in glial cells and neurons, where it facilitates free cholesterol (90%) and phospholipid (10%) efflux to ApoE, leading to the formation of high-density lipoprotein (HDL)-like ApoE-containing particles enriched with phospholipids and unesterified cholesterol [22]. Polymorphisms in the *ABCA1* gene have been linked to Alzheimer’s disease (AD) through disrupted cholesterol homeostasis [20], prompting extensive investigations of the transporter in the CNS.

To study the function of ABCA1 in vivo, several knockout mouse lines have been generated. Homozygous global ABCA1 knockout (ABCA1^−/−^) mice exhibit drastically reduced plasma HDL and diminished Apolipoprotein A-I (ApoA-I) levels, a cholesterol acceptor functionally analogous to ApoE [23]. In neuron- and glia-specific ABCA1 knockout mice (ABCA1^−B/−B^), marked reductions in plasma HDL cholesterol levels were observed, along with enhanced uptake of esterified cholesterol from blood plasma [24]. Affected mice showed reduced spontaneous locomotor activity and diminished sensorimotor functions, further demonstrating the impact of cholesterol on neural function. Deficiency of ABCA1 in the CNS is also associated with reduced oligodendrogenesis and compromised myelin repair after stroke injury [25]. Further, the absence of ABCA1 in astrocytes and microglia leads to reduced ApoE secretion, potentially by lysosomal degradation [26], and the accumulation of lipids within the cells [17,27]. Given the critical nature of cholesterol homeostasis in the nervous system, ABCA1 has been studied in a host of neurological disorders, including Alzheimer’s disease (AD), Parkinson’s and Huntington’s diseases, multiple sclerosis, epilepsy and stroke, among others [20]. With regards to myelination and myelin repair after ischemic injury, the redistribution of ABCA1 from lysosomes to the cell membrane was described as a key event for cholesterol transfer from microglia to oligodendrocytes [28]. While the involvement of ABCA1 in peripheral nerve remyelination has not been examined, the observed activation of this cholesterol transporter after nerve transection injury and the linkage of ABCA1 to hereditary peripheral nerve disorders support this possibility [16,18,29,30].

ABCA2, another member of the ABC family of transporters, is highly expressed in the brain, with a prominent abundance in oligodendrocytes [31]. Systemic ABCA2 knockout mice display neurological symptoms, including hind leg tremors and a shaking phenotype, and when analyzed, affected nerves contained thickened myelin, with atypical compaction [21,32]. Unlike the membrane association of ABCA1, the subcellular localization of ABCA2 is mostly endolysosomal, with roles in intracellular lipid trafficking, including cholesterol and sphingolipid homeostasis [21,33]. In the PNS, ABCA2 has been detected in both non-myelinating and myelin-forming Schwann cells, as well as vestibular schwannomas, which are benign glial cell tumors [34,35]. Nonetheless, the specific role of ABCA2 in peripheral nerve function and in Schwann cells has not been investigated, but based on its expression during early postnatal development it is postulated to include myelination [36]. Overall, ABCA2 has been proposed as a plausible therapeutic target in cardiovascular and Alzheimer’s diseases, and cancer [21]; however, because of its pleiotropic function, targeting its role in specific tissues and/or cell types poses challenges and will require further investigations.

## 3. Peripheral Neuropathy in Tangier Disease (TD)

Nearly thirty years ago, *ABCA1* mutations were identified as the cause of TD, a rare autosomal recessive disorder characterized by hepatosplenomegaly, peripheral neuropathy, and early-onset coronary artery disease [37]. Patients diagnosed with TD have a deficiency of high-density lipoproteins (HDLs) and show cholesterol accumulation within affected organs, including neural cells [16,38] (Table 1). At the cellular level, the expression of ABCA1 is reduced and affected tissues are unable to efflux cholesterol to Apolipoprotein A-1 acceptors and generate HDL particles [39]. When newly synthesized apolipoproteins cannot acquire cellular lipids via ABCA1, they are degraded or excreted, causing cholesterol to accumulate within cells, most notably in macrophages [40]. Due to cholesterol accumulation in various tissues, patients diagnosed with TD may present with orange–yellow tonsils, corneal opacity, and lymphadenopathy [38]. The subcellular defects due to ABCA1 misexpression were originally established in human skin fibroblasts from TD patients, which revealed defective endocytic trafficking and cholesterol retention in perinuclear vesicles [41]. While the genetic cause of TD has been investigated for three decades, specific therapies for affected patients have not been developed. Current recommendations include dietary modifications and lipid-controlling medications to reduce the risk of cardiovascular complications [40].

Neuropathic complications are frequent with TD and have been classified into four subtypes, including syringomyelia-like neuropathy (SMLN), multifocal mono- or polyneuropathy, and distal symmetric polyneuropathy (DSP) [16]. Nerve biopsy findings from affected individuals include the loss of myelinated axons, excessive lipid accumulation in Schwann cells, and endoneurial collagenous deposits [42,43]. In a rare nonsense mutation subtype of TD, sural nerve biopsies revealed severe neuropathy with the accompanied progressive loss of nerve fibers and pronounced endoneurial sclerosis [44]. In another clinical study of patient nerve biopsies, the noncompacted myelin region of the paranode (area adjacent to the node of Ranvier) was suggested to be a preferential site for atypical lipid accumulation [45]. The observed nerve pathology suggests that the space-occupying effects of the cholesterol esters contribute to paranodal malfunction and the formation of tomaculae, which are focal, sausage-shaped myelin thickenings [45]. Tomaculae are also hallmark features of nerves from patients with hereditary neuropathy with pressure palsy (HNPP), an inherited neuropathy associated with haploinsufficiency in PMP22 [46]. The overlapping nerve histopathology among these two distinct genetic disorders, TD and HNPP, suggest shared pathophysiology leading to a neuropathic phenotype. This association supports the partnership of ABCA1 and PMP22 in cholesterol metabolism, including lipid trafficking, within myelinated Schwann cells [29].

**Table 1:** *ABCA1* alterations and cholesterol mis-localization in *PMP22*-related hereditary peripheral neuropathies.

Disease and Genetic Cause	Effect on ABCA1 Expression	Fate of Cholesterol	Neurological Symptoms	References
Tangier Disease (TD): Mutations in *ABCA1*	Loss of functional protein	Severely reduced circulating HDL and cholesterol ester deposition in various cell types	Peripheral neuropathy: impaired nerve conduction, abnormal myelin	[16,39,45]
Charcot–Marie–Tooth type 1A (CMT1A): Duplication of *PMP22* gene	Unknown	Cholesterol sequestration to lysosomes	Demyelinating peripheral neuropathy including motor and sensory loss	[5,47,48]
Charcot–Marie–Tooth type 1E (CMT1E): Mutations in *PMP22*	Protein levels are elevated	Retained in ER–Golgi and reduced amounts in myelin	Demyelinating peripheral neuropathy, impaired nerve conduction	[30]
Hereditary Neuropathy with Pressure Palsies (HNPP): Deletion of *PMP22*	mRNA and protein levels are elevated	Reduced cholesterol in plasma membrane and accumulation in Golgi	Episodic, compression-induced neuropathy	[47]

## 4. Apolipoprotein E (ApoE) in Peripheral Nerve Biology

ApoE is a critical partner for ABCA1 within the nervous system and plays a vital role in cholesterol transport [49]. Within the CNS, ABCA1 facilitates the transfer of cholesterol from astrocytes onto ApoE and ApoA1, forming HDL particles [20]. The ApoE ε4 genotype has been associated with altered differentiation of oligodendrocytes and impaired myelination [50]. In accordance, in a recent ApoE allelic switch model, the replacement of Apo ε4 with the E2 allele exclusively in microglia improved post-injury remyelination, supporting a beneficial role for ApoE2 in CNS myelin [51].

In the PNS, ApoE is expressed by Schwann cells and was identified as a potential biomarker in sensory nerve fascicles, with its deficiency leading to sensory dysfunction and delayed thermal response [52]. The role of ApoE in peripheral nerve injury and regeneration remains unclear, however, with conflicting results in the literature. During nerve repair, following a crush injury, ApoE expression increases 100-fold, and declines once the regeneration is complete [13]. Consistent with a regenerative role, interventions with ApoE-mimetic peptides such as COG112, designed from the N-terminal of ApoE, significantly improved motor and sensory functional recovery, along with increased myelin thickness in repaired nerves [11]. Specifically, two weeks of daily intraperitoneal injection of an ApoE-mimetic peptide promoted axonal regeneration and remyelination and motor function in 8-week-old mice following sciatic nerve crush injury [11]. On the contrary, alternative studies using ApoE knockout (KO) mice did not reveal a significant difference in nerve regeneration following mechanical crush injury compared with wild-type (WT) animals, suggesting that ApoE may not be required for repair following this specific injury [53]. In another study, ApoE-null mice displayed impaired reinnervation and functional recovery following ischemic injury, with a delayed response to thermal stimuli and PNS sensory defects [54]. Moreover, re-expression of the human ApoE ε4 isoform in ApoE-null mice disrupted neuromuscular junction reinnervation after nerve injury compared to the ε3 isoform [55]. Together, these findings indicate that ApoE may contribute to peripheral nerve regeneration and remyelination in an isoform-dependent manner.

Although ApoE has been shown to support nerve repair in acute injury models, its potential as a therapeutic agent in chronic hereditary neuropathies remains unexplored. There is evidence to suggest that ApoE genetic variants may influence neuropathy severity. For instance, one study reported that carriers of the ApoE ε4 allele have a five-fold-increased risk of developing severe peripheral neuropathy when diagnosed with type II diabetes mellitus [56]. While these findings point to a possible modulatory role for ApoE in peripheral nerve vulnerability, there are no studies that have directly evaluated ApoE or ApoE-mimetic peptides as therapeutic interventions in inherited neuropathies, such as CMT or TD.

## 5. Myelin Defects in Charcot–Marie–Tooth Peripheral Neuropathies

Charcot–Marie–Tooth (CMT) diseases are the most common inherited neuropathies, characterized by progressive distal muscle weakness and atrophy [57], which can lead to deformities requiring surgery. CMT neuropathies encompass over one hundred genetic variations, giving rise to a wide range of subtypes. These neuropathies are typically classified based on inheritance pattern: autosomal dominant, x-linked, and autosomal recessive. Electrophysiological analysis further divides CMTs into two major types: type 1 (CMT1) and type 2 (CMT2). CMT1 is associated with significantly reduced nerve conduction velocities, indicating demyelination, whereas CMT2 is marked by decreased muscle action potentials, reflecting axonal degeneration [58].

In CMT type 1 monogenic peripheral neuropathies, the disease process originates in Schwann cells due to mutations in genes and gene products critical for myelin structure and function. These include *PMP22* (as seen in CMT1A, CMT1E, and HNPP), myelin protein zero (MPZ) (in CMT1B), and Gap Junction protein Beta 1 (GJB1), which encodes for the connexin 32 protein (in CMTX1) [1]. Given the essential role of cholesterol in maintaining the electrical and structural properties of cellular membranes, a number of these myelin-associated proteins have been investigated for their interaction with cholesterol. Notably, PMP22, along with myelin-associated glycoprotein (MAG), MPZ, and plasmolipin, harbor conserved Cholesterol Recognition Amino Acid Consensus (CRAC) motifs, signifying the importance of protein–lipid interaction and cholesterol stabilization in myelin [7]. In addition, cholesterol has been shown to be essential for the proper intracellular trafficking of MPZ, which constitutes nearly 50% of the total protein content in peripheral myelin [59].

Discrete defects of the *PMP22* gene give rise to individual CMT subtypes and clinical phenotypes, each impacting cholesterol homeostasis [5,60]. PMP22 is a 22 kDa hydrophobic, dosage-sensitive transmembrane glycoprotein whose under- and overexpression cause diseases [61]. Overexpression of PMP22 results in aberrant cholesterol accumulation and impaired lipid trafficking within Schwann cells, which are critical processes for effective myelin assembly [47]. A heterozygous duplication of the *PMP22* gene on chromosome 17p11.2 leads to CMT1A and represents the most prevalent form of CMT [62] with about 60,000 patients in the USA alone. This autosomal dominant mutation disrupts cholesterol homeostasis in Schwann cells by dysregulating key genes involved in the lipid transcription networks, resulting in dysfunctional myelin with reduced levels of cholesterol and ceramides [48]. Clinically, CMT1A patients typically present with distal muscle weakness and atrophy, hyporeflexia, and decreased sensory function [63]. In contrast, a heterozygous deletion of *PMP22* in the same genomic region causes HNPP, a less severe neuropathy [46]. HNPP is an autosomal dominant disease characterized by episodes of focal numbness, muscular weakness, and atrophy during adolescence. HNPP is also known as tomaculous neuropathy, due to the presence of sausage-like myelin thickening in affected nerves, similar to those described in TD [64].

A rare form of CMT, known as CMT1E, arises from point mutations in *PMP22*, instead of a gene duplication or deletion. Although the pathogenic mechanisms remain incompletely understood, CMT1E typically presents in infancy, often accompanied by additional features such as hearing loss, scoliosis, and hip dysplasia [65]. Much of what is known about the CMT1E pathophysiology has been derived from Trembler-J mice, which carry analogous point mutations in *PMP22* and exhibit profound demyelination and motor deficits [66,67]. CMT type 1B results from mutations of the most abundant structural protein in peripheral myelin with a CRAC domain, MPZ, whose gene is found on chromosome 1q22-q23 [63]. Mutations in MPZ lead to demyelinating neuropathy, disrupting myelin compaction and Schwann cell function [68]. In each of these inherited neuropathies, myelin is abnormal, which could be the result of aberrant cholesterol trafficking, distribution and presentation within myelinating Schwann cells [60]. As shown in Table 1, in addition to TD-associated peripheral neuropathy, cholesterol mis-localization and altered ABCA1 expression have been observed in type 1 neuropathies linked with *PMP22*. These commonalities across the four distinct diseases suggest shared pathophysiology that could benefit from ABCA1-targeted therapies.

## 6. Considerations in Targeting ABCA1 in Neurological Disorders to Remedy Cholesterol Mis-Localization

Due to its critical role in cholesterol metabolism, ABCA1 has emerged as a therapeutic target in both cardiovascular and Alzheimer’s disease (AD) [69,70,71]. AD-related pathology and cognitive impairment have been alleviated through ABCA1 upregulation by liver X receptor (LXR) or retinoid X receptor (RXR) agonists, as demonstrated in multiple transgenic mouse models [72]. In APP23 transgenic mice, LXR activation reduced amyloidogenic APP processing and Aβ production [73], along with a reduction in cognitive deficits [74]. These effects were associated with increased ABCA1 expression, highlighting functional ABCA1 induction as a promising therapeutic target for AD [73,74,75]. Unfortunately, the use of LXR and RXR agonists in humans is associated with often-severe side effects, including hepatomegaly and extreme hypertriglyceridemia deemed to disqualify their use in human chronic conditions [76,77].

An alternative promising approach for correcting atypical cholesterol metabolism is the employment of CS6253, an ABCA1 agonist peptide [69]. CS6253 is designed from the lipid-binding C-terminal of ApoE and optimized for ABCA1-mediated cholesterol efflux [71]. CS6253 has direct ABCA1 effects and does also, by competing with ApoE and ApoA-I binding to HDL, generate smaller HDL particles and increased plasma ApoE [69]. CS6253 stabilizes ABCA1 and prevents protein degradation leading to increased ABCA1 concentration and enhanced cholesterol efflux to extracellular ApoE acceptor particles. Notably, CS6253 has demonstrated efficacy in improving disease phenotypes in models of AD [78,79,80] and multiple sclerosis [81,82]. For example, when CS6253 was administered intraperitoneally for six weeks to 2.5-month-old male mice expressing human ApoE3 or ApoE4, the peptide intervention in ApoE4-targeted replacement (TR) mice upregulated ABCA1 and increased lipidation of ApoE particles in the brain. In addition, CS6253 treatment increased circulating plasma ApoE, aligning ApoE4 TR mice more closely with ApoE3 TR mice regarding ABCA1 and ApoE levels and composition [78]. Further, CS6253 reduced Aβ42 accumulation and tau hyperphosphorylation in hippocampal neurons, improved cholesterol transport, and counteracted cognitive decline [78]. In human astrocytes expressing ApoE4, ABCA1 protein levels are reduced, resulting in impaired cholesterol efflux [83]. Thus, upregulating ABCA1 and subsequently enhancing ApoE lipidation may represent a viable therapeutic strategy for neurodegenerative diseases characterized by disrupted lipid homeostasis. Significantly, CS6253 has undergone IND-enabling studies in rats and cynomolgus monkeys showing a wide safety margin between effective and toxic doses (data on file). Under an open IND, a rigorous Phase 1 Single Ascending Dose, Multiple Ascending Dose, and subcutaneous bioequivalence study in 66 healthy men and women, the majority 50 years and older (NCT05965414), CS6253 showed favorable safety and pharmacokinetics, and in addition, biomarker effects including transient increases in plasma small HDL, ApoE, and signals of amyloid clearance [78,84,85]. At the cellular level, CS6253 treatment is associated with increased ABCA1 localization to the cell membrane [26,79], reducing the free cholesterol in the membrane, potentially affecting the lipid raft architecture and function of transmembrane proteins such as ABCA1 and PMP22.

**Figure 1:**
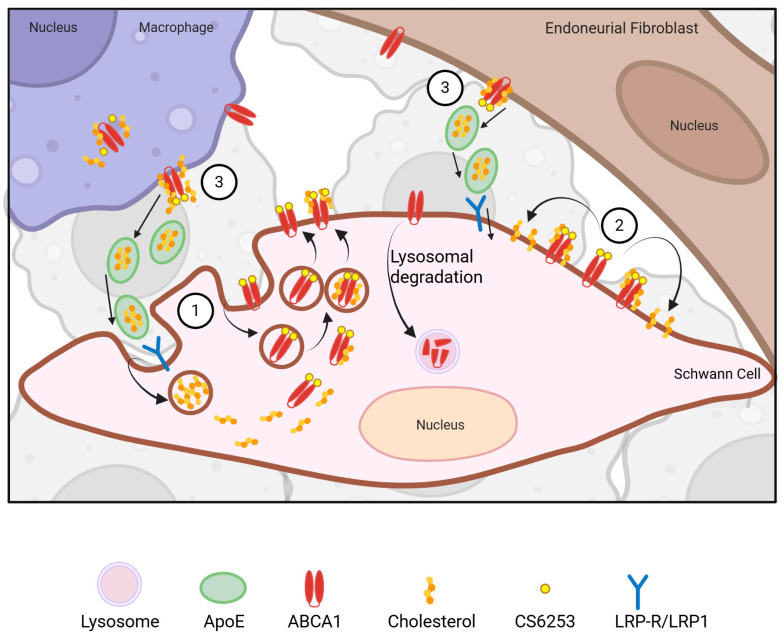
Potential mechanisms by which CS6253 administration may modulate ABCA1 function and cholesterol homeostasis in the peripheral nervous system. CS6253 is proposed to bind ABCA1 at the Schwann cell membrane (CM), activating vesicular recycling of ABCA1 together with cholesterol. This process may increase intracellular trafficking of ABCA1 and cholesterol and promote redistribution of atypical intracellular cholesterol back to the CM, thereby restoring CM cholesterol content and organization (1). Second, CS6253 may activate ABCA1 on the cell surface of macrophages and/or endoneurial fibroblasts, thereby activating cholesterol efflux for uptake through LDL-R-mediated mechanism by neighboring Schwann cells (2). Further, CS6253 may bind ABCA1 on the Schwann cell surface, and redistribute cholesterol in the cell membrane (3). Created in BioRender. Woo, Y. (2026) https://biorender.com/njg45kn.

Given the knowledge that activation of ABCA1 can elicit a positive response in neurological disorders with cholesterol transport pathophysiology, ABCA1 becomes a feasible target in peripheral neuropathies. This includes, for example, severe forms of type 1 neuropathies such as HNPP and CMT1E, in which ABCA1 expression along with ApoE are upregulated, likely as a compensatory mechanism for defective cholesterol transport [29,86]. Interestingly, ABCA1 was mis-localized intracellularly in neuropathic nerves, as opposed to normal membrane-associated localization. In parallel with the ABCA1 mis-localization, we also detected abnormal clumping and sequestration of cholesterol [29]. Further, the complete absence of PMP22 impaired the cholesterol efflux capacity of Schwann cells, indicating a deficiency in ABCA1 functionality at the plasma membrane. The compensatory ABCA1 upregulation to the absence of PMP22 was therapeutically insufficient as the animals developed severe myelin defects. Conversely, in nerves from ABCA1 knockout mice, a model of TD, the expression of PMP22 was significantly elevated, and the subcellular processing of the overproduced PMP22 was compromised. The shared tomaculous neuropathic phenotypes in humans and animals with altered PMP22 or ABCA1 expression indicate a common cholesterol transport pathophysiology. We hypothesize that, in PNS diseases with underlying myelin defects, there is a fundamental impairment in PMP22-ABCA1 protein–protein interaction, leading to atypical cholesterol transport and localization. ABCA1-targeting therapies can potentially counter the PMP22-ABCA1 functional impairment by correcting the perturbed cholesterol homeostasis in the plasma membrane, including lipid rafts [86]. This hypothesis is supported by data from mouse models where ABCA1 upregulation by CS6253 administration improved CNS myelin content and the disease phenotype [81,82]. Taken together, these findings support the therapeutic potential of ABCA1 agonist peptides such as CS6253 for enhancing cholesterol mobilization in neuropathic Schwann cells, correcting cell membrane abnormalities, and facilitating myelin repair. Activation of ABCA1, even in the absence of PMP22, such as in severe HNPP, has therapeutic potential, as indicated in the hypothetical working model (Figure 1).

Support for the beneficial effects of ABCA1 activity on myelin and membrane expansion can be derived from independent basic cellular studies. The recycling and exchange of lipids within a neuropathic nerve can be envisioned between resident macrophages, endoneurial fibroblasts and Schwann cells, all of which are known to express ABCA1 (see Figure 1) [29,87]. A similar mechanism was recently observed in a study of glioblastoma, where macrophage-mediated myelin recycling was shown to provide metabolic support to dividing cells, in effect promoting glioblastoma progression, suggesting potential for ABCA1 targeting [87]. A third, less-studied process by which the activation of ABCA1 could improve myelination is through cholesterol redistribution within the Schwann cell plasma membrane [88], and regulation of cholesterol content and membrane properties of the endoplasmatic reticulum and possibly other organelles [89]. The proposed hypothetical working model in Figure 1 depicts these possibilities and awaits experimental confirmation.

Each of these scenarios could enhance cholesterol recycling/redistribution and restore Schwann cell membrane lipid homeostasis in neuropathic conditions.

## 7. Conclusions and Future Directions

Despite growing recognition of cholesterol’s critical role in peripheral nerve function, the mechanisms regulating its intracellular transport and membrane distribution within myelinated nerves remain poorly understood. The common cellular pathophysiology of tomaculous neuropathy in TD and HNPP, however, suggest a shared susceptibility of myelin to disrupted cholesterol trafficking and localization. One promising therapeutic strategy involves engaging ABCA1 to restore lipid homeostasis in affected nerves. Targeting ABCA1, whether through nuclear receptor agonists or ABCA1 agonist peptides such as CS6253, has shown potential to correct abnormal cholesterol distribution and support proper remyelination in the CNS. Further studies should investigate the impact of ABCA1 agonists to assess their ability to normalize myelin structure and improve function in peripheral nerves and at nerve-muscle junctions. Advancing our understanding of the role for ABCA1 in cholesterol trafficking and myelination in Schwann cells may open the door to new therapies for a wide range of peripheral nerve disorders, including CMTs, painful facial neuropathies such as Trigeminal Neuralgia, and other polyneuropathies.

## Data Availability

No new data were created or analyzed in this study. Data sharing is not applicable to this article.
